# Shp2 Deficiency in Kupffer Cells and Hepatocytes Aggravates Hepatocarcinogenesis by Recruiting Non-Kupffer Macrophages

**DOI:** 10.1016/j.jcmgh.2023.02.011

**Published:** 2023-02-23

**Authors:** Li Du, Yichun Ji, Bing Xin, Jiemeng Zhang, Li-Chun Lu, Christopher K. Glass, Gen-Sheng Feng

**Affiliations:** 1 Department of Pathology, Department of Molecular Biology, Moores Cancer Center, University of California San Diego, La Jolla, California; 2 Department of Cellular and Molecular Medicine, University of California San Diego, La Jolla, California; 3 Department of Gastroenterology, Union Hospital, Tongji Medical College, Huazhong University of Science and Technology, Wuhan, China; 4 Department of Oncology, National Taiwan University Hospital, Taipei, Taiwan

**Keywords:** Hepatocyte/Kupffer Cell Communication, Hepatocarcinogenesis, Tumor-Associated Macrophages, Tumor Microenvironment

## Abstract

**Background & Aims:**

Complex communications between hepatocytes and Kupffer cells (KCs) are known to drive or suppress hepatocarcinogenesis, with controversial data in the literature. In previous experiments that aimed to decipher hepatocyte/KC interactions, we unexpectedly unveiled a tumor-suppressing effect of polyinosinic-polycytidylic acid, a widely used inducer of MX dynamin like GTPase 1 (Mx1)-cre expression, which questioned a theory of interleukin 1a/6 cytokine circuit in hepatocyte/KC communication. The goal of this study was to clarify the controversy and decipher unique functions of KCs and non-KC macrophages in liver tumorigenesis.

**Methods:**

We used the C-type lectin domain family 4 member F (Clec4f)-cre system to delete Src-homology 2 domain-containing tyrosine phosphatase 2 (Shp2)/protein tyrosine phosphatase nonreceptor 11 (Ptpn11) in KCs, and a combination of Clec4f-cre and adeno-associated virus–cre to delete Shp2 in KCs and hepatocytes to investigate the effects on hepatocellular carcinoma development and immune cell compositions/activities.

**Results:**

Ablating Shp2 in KCs generated a tumor-promoting niche, which was exacerbated further by concurrent removal of Shp2 in both KCs and hepatocytes. Shp2 deficiency induced KC apoptosis and decreased its numbers, which induced compensatory recruitment of bone marrow–derived monocytes into liver. These newly recruited monocytes differentiated into non-KC macrophages with tumor-associated macrophage function, leading to aggravated tumor progression through down-regulation of CD8 T cells. Tumor-associated macrophage blockade by anti-chemokine (C-C motif) ligand 2 (CCL2) antibody inhibited hepatocellular carcinoma progression, while depletion of all macrophages had a tumor-promoting effect by increasing myeloid-derived suppressor cells (M-MDSCs) and decreasing CD8 T cells.

**Conclusions:**

Shp2 loss in KCs or hepatocytes generated a protumorigenic microenvironment, which was exacerbated by its removal in both cell types. These results show the complexity of intercellular signaling events in liver tumorigenesis and raises caution on the use of specific Shp2 inhibitor in liver cancer therapy. Transcript profiling: RNA sequencing data are available at Gene Expression Omnibus (GSE222594).

**Summary**

Our data show that deleting Src-homology 2 domain-containing tyrosine phosphatase 2 (Shp2) in Kupffer cells enhances hepatic recruitment of monocyte-derived macrophages and a tumor-promoting niche. Given that Shp2 is currently a very popular drug target, this study raises caution on targeting Shp2 in liver cancer therapy.

Hepatocellular carcinoma (HCC) has become a most deadly malignant disease worldwide.^1^ For patients with early stage HCC, local ablation and surgical resection often are applied for curative treatment.^2^ However, HCC recurred in more than half of these patients within 2 years, and rapidly progressed into advanced stages.^3^ In addition, a significant population of HCC patients initially were diagnosed at advanced stages, owing to a lack of clinical symptoms at early stages.^4^ Treatment options for advanced HCC are limited, with liver transplantation remaining a primary choice.^2^

In-depth understanding of molecular and cellular mechanisms that drive HCC development will be instrumental for design of targeted therapeutic strategies. Remarkably, several groups have shown that genetic ablation of classic oncoproteins such as inhibitor kappa-B kinaseβ (Ikkβ), inhibitor kappa-B kinaseβ 1/2 (Jnk1/2), epidermal growth factor receptor (EGFR), β-catenin, and protein kinase B (Akt) in hepatocytes exacerbated HCC developed spontaneously or induced by chemical carcinogen diethylnitrosamine (DEN),^5^, ^6^, ^7^, ^8^, ^9^ disclosing complex mechanisms of liver tumorigenesis. In contrast, HCC development was suppressed by deletion of Ikkβ, Jnk1/2, and EGFR in both hepatocytes and Kupffer cells (KCs), using the Mx1-cre system induced by polyinosinic-polycytidylic acid (polyIC).^6^^,^^8^^,^^9^ These bidirectional effects of signaling molecules led to a proposal that loss of these prosurvival molecules in hepatocytes induced hepatocyte production of interleukin (IL)1a, which stimulated macrophages to produce IL6 that in turn promoted hepatocyte proliferation and transformation, resulting in more severe HCC development.^6^ The communication between hepatocytes and KCs via a cytokine circuit of IL1α/IL6 was disrupted by simultaneous removal of the signaling molecules in hepatocytes and macrophages by Mx1-cre.

In previous experiments, we observed that hepatocyte-specific deletion of Src-homology 2 domain-containing tyrosine phosphatase 2 (Shp2) aggravated DEN-induced HCC, similar to the effects of Ikkβ, Jnk1/2, or EGFR removal from hepatocytes. Nevertheless, by using the MX dynamin like GTPase 1 (Mx1)-cre mouse line to delete Shp2, we surprisingly unveiled a tumor-inhibiting effect of polyIC, the reagent used to induce Mx1-cre expression, independent of Shp2 deletion in hepatocytes and KCs.^10^ These results call for re-interpretation of the previous data on HCC suppression observed with the inducible Mx1-cre system for gene deletion. Of note, the Mx1-cre system with polyIC induction still is being used in liver cancer research,^11^^,^^12^ but the results could be misleading because of the complicated antitumorigenic or protumorigenic effects of the synthetic double-stranded RNA (dsRNA) that induces a milieu of inflammatory cytokines.

In the present study, we used the Clec4f-cre system to delete Shp2/protein tyrosine phosphatase nonreceptor 11 (Ptpn11) in KCs,^13^ and combination of Clec4f-cre and adeno-associated virus (AAV)-cre to delete Shp2 in KCs and hepatocytes. We show that selective deletion of Shp2 in KCs down-regulated the KC pool and enhanced hepatic recruitment of bone marrow–derived monocytes, which differentiated into non-KC macrophages. Strikingly, Shp2 loss in KCs and/or hepatocytes aggravated primary and metastatic liver tumor progression owing to accumulation of tumor-promoting macrophages and suppression of CD8 T lymphocytes. These results, although challenging the previous theory on hepatocyte–KC communication in driving HCC, also show multifaceted functions of macrophages in the liver, which may guide design of more effective liver cancer therapy.

## Results

### Shp2 Deletion Downsizes the KC Pool and Promotes Hepatic Recruitment of Bone Marrow–Derived Monocytes

**Figure 1:**
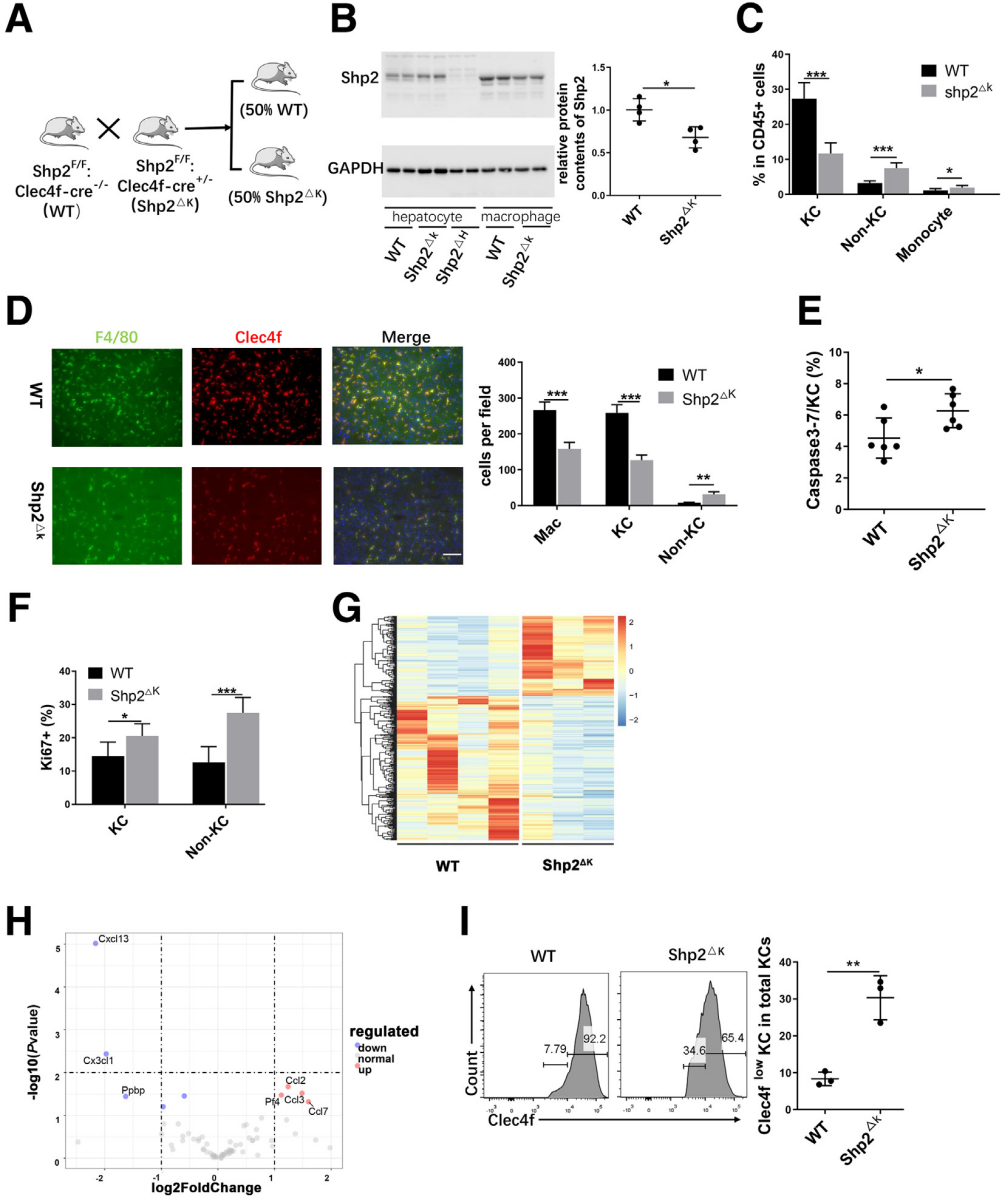
**Shp2 deletion downsizes the KC pool and induces hepatic recruitment of bone marrow-derived macrophages**. (*A*) Experimental scheme to generate a mutant mouse line Shp2^ΔK^ (Shp2^F/F^:Clec4f-cre^+/-^). (*B*) *Left*: Representative immunoblotting for Shp2 protein in hepatocytes and macrophages isolated from 2-month-old mouse livers of various genotypes. *Right*: Relative Shp2 protein levels in isolated macrophages of WT and Shp2^DK^ livers. (*C*) FACS analysis to quantify the percentages of KCs (Clec4f^+^ CD11b^+^ F4/80^high^), non-KC macrophages (Clec4f^-^ CD11b^+^ F4/80^high^), and monocytes (CD11b^+^ Ly6C^+^) in CD45^+^ cells in mouse livers at 2 months. Data are presented as means ± SD (n = 6). (*D*) Representative immunostaining (*left*) and quantification (*right*) of total macrophages (Mac, F4/80^+^), KCs (F4/80^+^ Clec4f^+^), and non-KC macrophages (F4/80^+^ Clec4f^-^) in liver sections. Magnification, ×200; *scale bar*: 50 μm. (n = 5 per group) (*E*) FACS analysis to quantify the ratios of caspase-3/7 cells in KCs. (*F*) FACS analysis to show the ratios of Ki67^+^ cells in KCs and non-KC macrophages (n = 6). (*G*) A heatmap built with RNA-seq data shows the different gene expression profiles in NPCs isolated from WT and Shp2^ΔK^ livers at age 2 months. (*H*) Volcano plot for up-regulated and down-regulated expression of chemokines and adhesion molecules in NPCs of Shp2^ΔK^ livers, relative to WT controls. (*I*) Representative FACS analysis and quantification to show the ratios of Clec4f^low^ KCs in total KCs in WT and Shp2^ΔK^ livers. ∗*P* < .05; ∗∗*P* < .01; ∗∗∗*P* < .001. GAPDH, glyceraldehyde-3-phosphate dehydrogenase.

**Figure 2:**
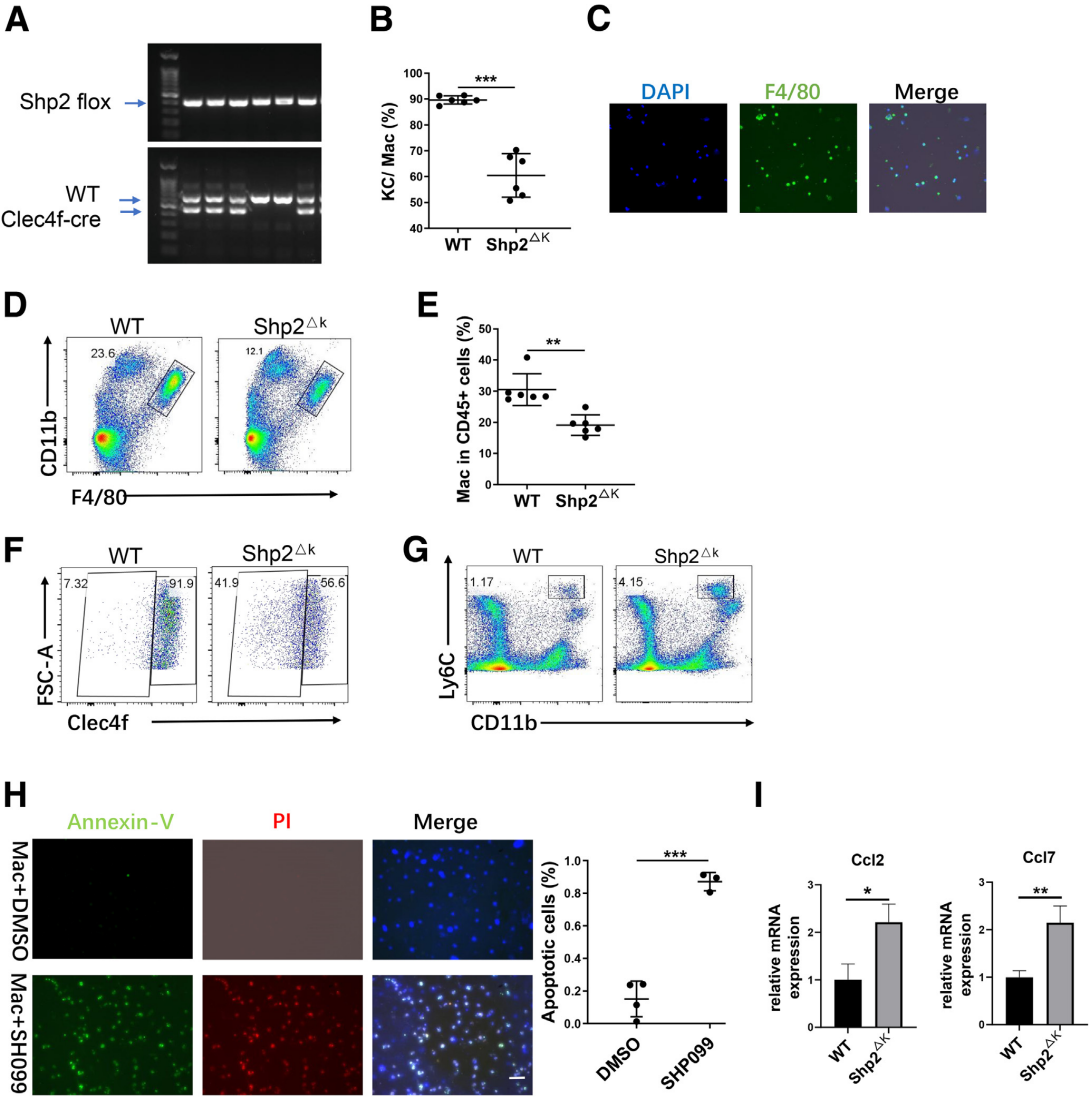
**Representative flow cytometric images to show macrophages, KCs, and monocytes.** (*A*) PCR was performed to determine the genotypes of Shp2^flox^ and Shp2^WT^ alleles as well as the Clec4f-cre transgene. (*B*) FACS analysis to quantify ratios of KCs in total macrophages in livers. (*C*) Representative immunostaining of F4/80^+^ cells in isolated macrophages in the liver. (*D*) Representative flow cytometric images to show macrophages (CD11b^+^ F4/80^high^) in CD45 cells in the liver. (*E*) FACS analysis to quantify ratios of macrophages in CD45^+^ cells in livers. (*F*) Representative flow cytometric images to show KCs (Clec4f^+^ macrophages) and non-KC macrophages (Clec4f^-^ macrophages) in total macrophages in the liver. (*G*) Representative flow cytometric image to show monocytes (CD11b^+^ly6C^high^) in CD45^+^ cells in the liver. (*H*) Annexin-V and propidium iodide staining for cell apoptosis induced by 1 μM (μmo/L) of SHP099. (*I*) Quantitative reverse-transcription PCR to determine expression of Ccl2 and Ccl7 mRNA in NPCs in mouse livers at 2 months (n=5 per group). ∗*P* < .05; ∗∗*P* < .01; ∗∗∗*P* < .001. DAPI, 4′,6-diamidino-2-phenylindole; DMSO, dimethyl sulfoxide; FSC-A, forward scatter-area; Mac, macrophage.

To define a functional role of Shp2 in KCs in the liver, we crossed Shp2^F/F^ mice with Clec4f-cre^+/-^ transgenic mice to generate a Shp2^F/F^:Clec4f-cre^+/-^ (Shp2^ΔK^) mouse line, with Shp2/Ptpn11 ablated in KCs (Figures 1*A* and 2*A*). Clec4f is a specific marker for KCs and not expressed in other macrophages in the liver and other organs.^13^ Of note, KCs are liver-resident macrophages that constitute nearly 90% of total macrophages in the liver (Figure 2*B*). We isolated liver macrophages (Figure 2*C*), and immunoblot analysis showed only a modest decrease of Shp2 protein amounts in macrophages of Shp2^ΔK^ mice, relative to WT control (Shp2^ΔK^/WT, 68.07%) (Figure 1*B*). It was shown previously that depletion of KCs by diphtheria toxin (DT) expression under control of Clec4f induced hepatic recruitment of monocytes, which differentiated first into non-KC macrophages and then new KCs for compensation.^13^ Because Shp2 is a positive regulator for cell proliferation,^14^ we asked if KC numbers decreased in the Shp2^ΔK^ mouse. The percentages of total liver macrophages decreased in Shp2^ΔK^ mice (Figure 2*D* and *E*), in which KCs decreased, while monocytes and non-KC macrophages increased (Figures 1*C* and 2*F* and *G*). The fluorescence-activated cell sorter (FACS) data were validated by immunostaining for F4/80 and Clec4f (Figure 1*D*). Because of restricted expression of Clec4f in KCs, Shp2 was not deleted in newly recruited non-KC macrophages, which explains the modestly reduced levels of Shp2 in the whole macrophage pool of Shp2^ΔK^ mice.

Shp2 deletion by Clec4f-cre promoted KC apoptosis in vivo (Figure 1*E*), as assessed by caspase 3/7 expression, and in cell culture, by Annexin-V staining (Figure 2*H*). Interestingly, proliferation of both KCs and non-KC macrophages was up-regulated in Shp2^ΔK^ mice (Figure 1*F*), in agreement with previous data showing higher KC proliferation in the first 2 weeks after DT-mediated KC depletion.^15^ We then measured messenger RNA (mRNA) levels of monocyte-associated chemokines and adhesion molecules to explore the underlying mechanisms by RNA-sequencing (RNA-seq) analysis of nonparenchymal cells (NPCs) isolated from WT and Shp2^ΔK^ livers (Figure 1*G*). The expression of chemokines Ccl2, Ccl3, Ccl7, and adhesion molecule pf4 were significantly higher in NPCs of Shp2^ΔK^ than WT liver (Figure 1*H*), and increased expression of Ccl2 and Ccl7 was confirmed by quantitative reverse-transcription polymerase chain reaction (PCR) data (Figure 2*I*). Moreover, 30.33% of KCs expressed lower levels of Clec4f in Shp2^ΔK^ mice, much higher than 8.33% in WT mice, suggesting that these were newly differentiated KCs in Shp2^ΔK^ liver (Figure 1*I*). Together, these data suggest that Shp2 deficiency induced KC apoptosis and decreased KC numbers, which triggered compensatory monocyte recruitment and non-KC macrophage/KC differentiation and proliferation in the liver. When the newly differentiated KCs started to express Clec4f-cre, it drove Shp2 deletion and then KC apoptosis, constituting a dynamic loop featured by persistent recruitment of monocytes and increased non-KC macrophages, to compensate for the KC deficit.

### Shp2 Deficiency in KCs Exacerbates Metastasized Liver Tumor Progression

**Figure 3:**
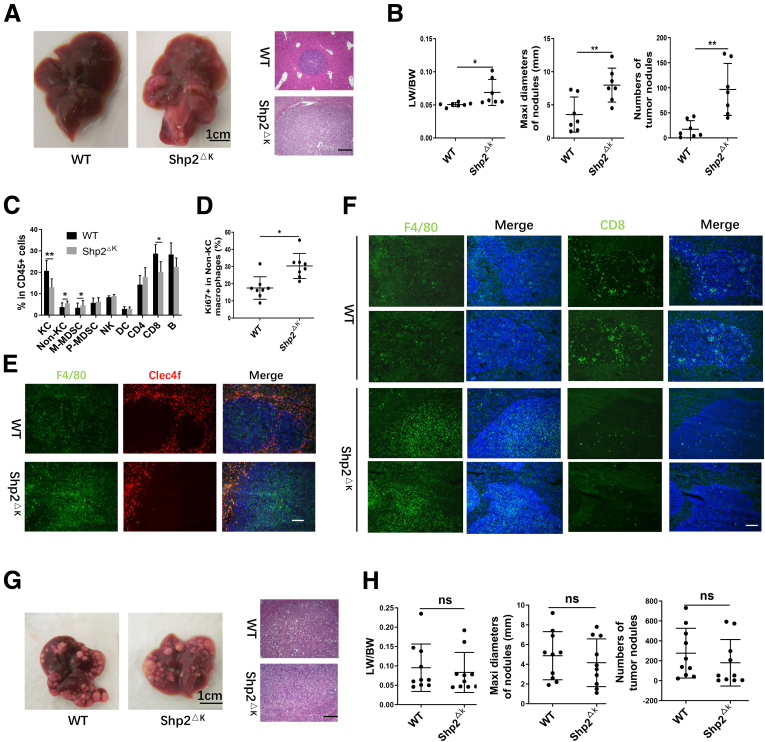
**Loss of Shp2 in KCs promotes metastatic liver tumor progression.** (*A*) Representative macroscopic views and H&E staining of tumor-bearing WT and Shp2^ΔK^ mouse livers 16 days after intrasplenic injection of Mouse Colon Cancer Cells 38. (*B*) Tumor burdens were evaluated by liver weight to body weight (LW/BW) ratios, maximal diameters, and number of tumor nodules. Data are presented as means ± SD. (*C*) FACS analysis to determine the ratios of various immune cell subsets in CD45^+^ cells in mouse livers 10 days after injection of Mouse Colon Cancer Cells 38. Data are presented as means ± SD (n = 8 per group). (*D*) FACS analysis to quantify the ratios of Ki67^+^ cells in non-KC macrophages. (*E*) Representative immunostaining of KCs (F4/80^+^ Clec4f^+^) and non-KC macrophages (F4/80^+^ Clec4f^−^) in liver sections. Magnification, ×100; *scale bar*: 100 μm. (*F*) Immunostaining of F4/80^+^ or CD8^+^ cells in tumor areas of liver sections. Magnification, ×100; *scale bar*: 100 μm. (*G*) Representative macroscopic views and H&E staining of liver sections 6 weeks after oncogene transfection. Magnification, ×100; *scale bar*: 200 μm. (*H*) Tumor burdens were evaluated by LW/BW ratios, maximal diameter, and number of tumor nodules. Data are presented as means ± SD (n = 10). ∗*P* < .05; ∗∗*P* < .01. Maxi, maximum.

Given that ablating Shp2 in KCs induced dynamic changes in compositions of the liver macrophage population, we reasoned that it might have a significant impact on the hepatic immune ecosystem and on liver tumorigenesis. To address this issue, we took 2 different approaches to evaluate hepatic responses to tumor growth in Shp2^ΔK^ mice. First, we examined metastasized liver tumors after intrasplenic injection of Mouse Colon Cancer Cells 38.^16^ When evaluated 16 days later by the numbers and sizes of tumor nodules and also the liver weight/body weight ratios, the tumor burdens were significantly higher in Shp2^ΔK^ mice than WT controls (Figure 3*A* and *B*). Then, we compared immune cell compositions between tumor-bearing WT and Shp2^ΔK^ mice, and detected significantly reduced numbers of KCs, increased non-KC macrophages and M-MDSCs, with a reduction of CD8 T cells (Figure 3*C*). The compensatory recruitment of monocytes and the increased proliferation rate apparently contributed to the higher percentages of non-KC macrophages (Figure 3*D*). Immunostaining further confirmed that tumor-associated macrophages (TAMs) were non-KC macrophages (F4/80^+^ and Clec4f^-^), a population expanded in Shp2^DK^ mice (Figure 3*E*). TAMs were reported to suppress CD8 T cells by inhibiting their proliferation and secreting extracellular matrix to exclude CD8 T cells from the tumor nest. Thus, we chose to examine TAMs and CD8 T cells in the same fields of liver sections, and found that the increased TAMs correlated well with reduced CD8 T cells in Shp2^DK^ mice (Figure 3*F*).

We also investigated the effect of Shp2 loss in KCs on tumorigenesis in a primary HCC model after hydrodynamic tail vein injection (HTVi) of 2 oncogenes, N-Rat sarcoma virus (N-Ras) and cellular-Myelocytomatosis (c-Myc), together with a sleeping beauty transposase construct, into WT and Shp2^ΔK^ mice.^17^ Tumor loads were evaluated 6 weeks after oncogene transfection. Interestingly, ablating Shp2 in KCs did not significantly influence the primary tumor growth, as assessed by the numbers and sizes of tumor nodules and the liver weight/body weight ratios (Figure 3*G* and *H*). Taken together, these results suggest that selective deletion of Shp2 in KCs generated a protumorigenic niche in the liver, which was sufficient to aggravate tumor progression from metastasized tumor cells, but was insufficient to promote initiation and development of primary liver cancer driven by the oncogenes Ras and Myc.

### Loss of Shp2 in KCs and Hepatocytes Aggravates Primary Liver Cancer Development

**Figure 4:**
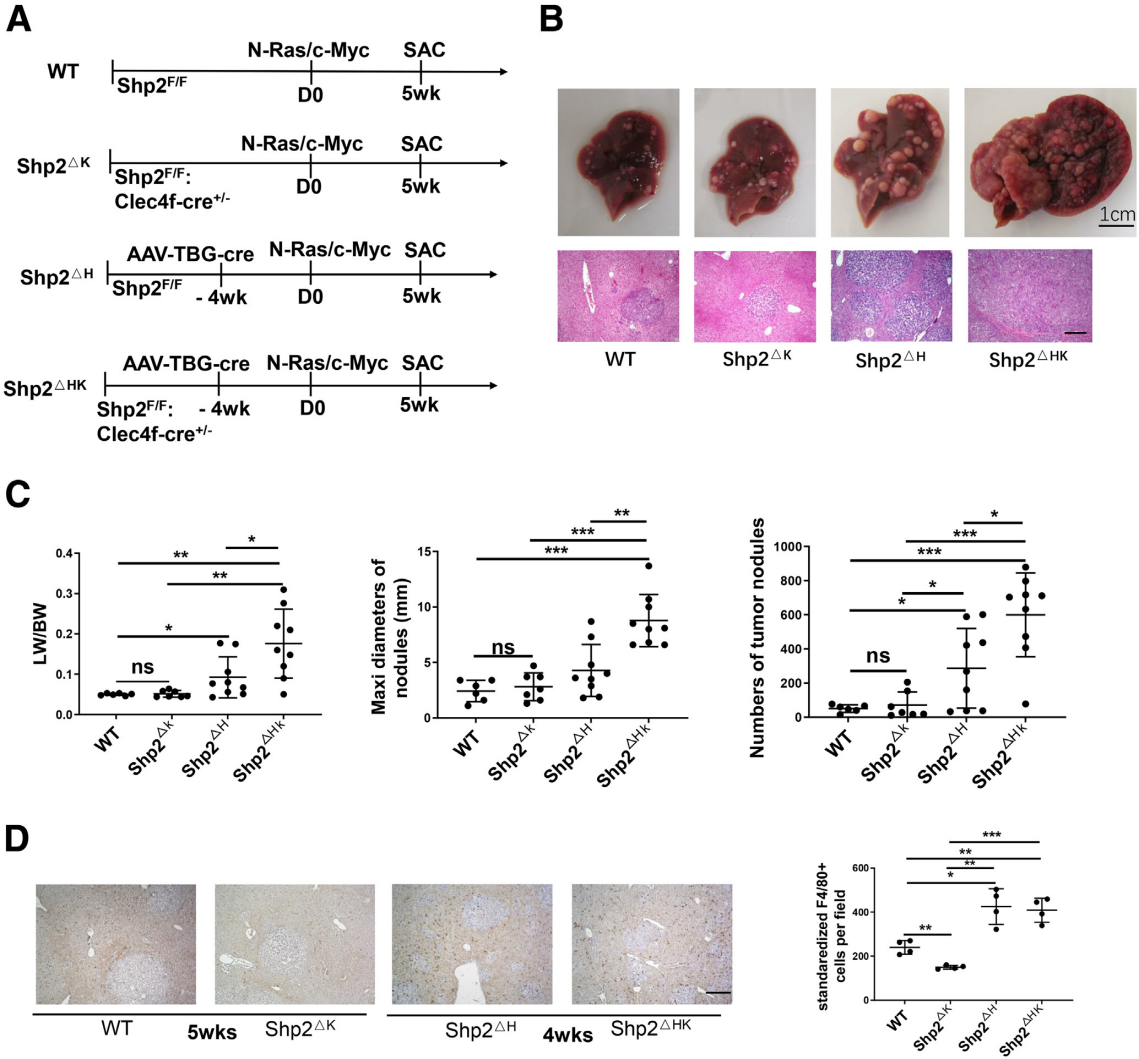
**Shp2 removal from KCs and hepatocytes promotes primary liver tumorigenesis.** (*A*) The experimental scheme to induce HCC by N-Ras/c-Myc oncogenes via HTVi into WT, Shp2^ΔK^, Shp2^ΔH^, and Shp2^ΔHK^ mice. (*B*) Representative macroscopic views and H&E staining of liver sections 5 weeks after oncogene transfection. Magnification, ×100; *scale bar*: 200 μm. (*C*) Tumor burdens were evaluated by liver weight/body weight (LW/BW) ratios, maximal diameters, and number of tumor nodules. Data are presented as means ± SD (n = 7–9 per group). (*D*) Representative immunostaining (*left*) and quantification (*right*) of F4/80^+^ cells in liver sections with similar tumor sizes at 4 or 5 weeks. Magnification, ×100; *scale bar*: 200 μm. ∗*P* < .05; ∗∗*P* < .01; ∗∗∗*P* < .001. Maxi, maximum; SAC, sacrifice; TBG, thyroxine-bindlng globulin.

We then asked if deleting Shp2 in both KCs and hepatocytes would affect HCC initiation and progression. To address this question, we injected AAV-Cre virus via tail vein into Shp2^F/F^ and Shp2^ΔK^ mice, to generate Shp2^ΔH^ and Shp2^ΔHK^ mouse lines with Shp2 deleted in hepatocytes and KCs plus hepatocytes, respectively (Figure 4*A*). We compared the tumor burdens between Shp2^ΔH^ and Shp2^ΔHK^ mice after transfection of Ras/Myc oncogenes using the HTVi approach. In agreement with our previous data,^17^ Shp2 loss in hepatocytes mediated by AAV-Cre aggravated Ras/Myc-driven tumor loads in Shp2^ΔH^ mice, relative to WT and Shp2^ΔK^ mice (Figure 4*B* and *C*). However, the tumor burdens were significantly higher in Shp2^ΔHK^ mice than in Shp2^ΔH^ mice (Figure 4*B* and *C*), suggesting that concurrent removal of Shp2 from both KCs and hepatocytes further promoted HCC progression. Shp2 loss in hepatocytes triggered active recruitment of macrophages into liver by comparing total macrophage numbers among WT, Shp2^ΔK^, Shp2^ΔH^, and Shp2^ΔHK^ mice (Figure 4*D*). Here, we chose tumors with similar sizes in each group for comparison to exclude the effect of tumor volumes on macrophage recruitment. Thus, Shp2 removal in KCs or hepatocytes generated a protumorigenic microenvironment, which was exacerbated by its concurrent deletion in both cell types. This result stands in contrast to the previous data that deleting pro-oncogenic molecules in both hepatocytes and KCs were tumor-suppressive.^5^

### Tumor-Promoting Myeloid Cell Subsets Are Accumulated in Shp2^ΔHK^ Mice

**Figure 5:**
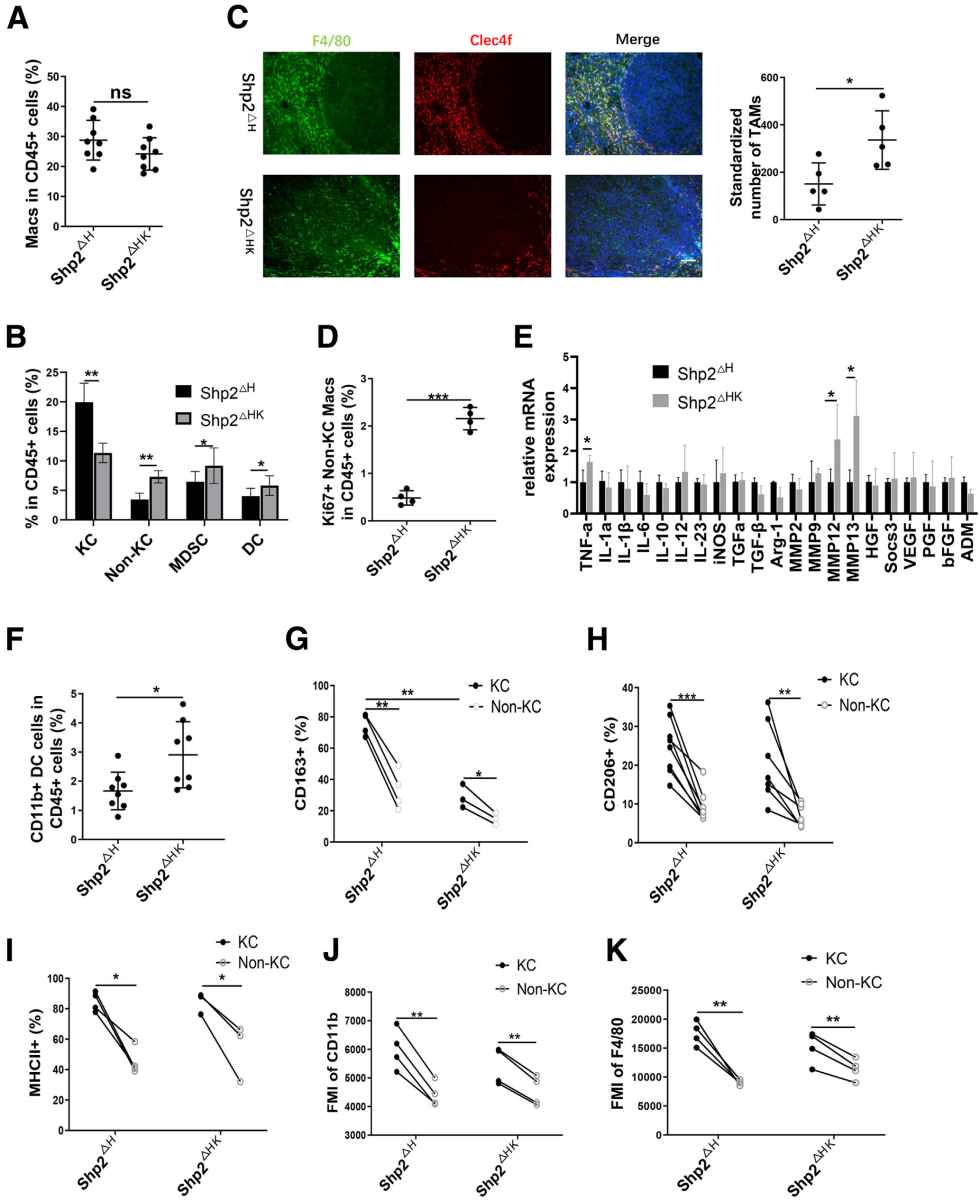
**Accumulation of hepatic myeloid cells in tumors of Shp2**^**ΔHK**^**mice.** (*A*) FACS analysis to evaluate ratios of total macrophages in CD45^+^ cells in the livers. (*B*) FACS analysis to determine the ratios of KCs, non-KCs, MDSCs, and DCs in hepatic CD45^+^ cells (n = 8). (*C*) Representative immunostaining and quantification of TAMs in the liver. Magnification, ×100; *scale bar*: 100 μm. (*D*) FACS analysis to quantify the ratios of Ki67^+^ non-KC macrophages in hepatic CD45^+^ cells. (*E*) Quantitative reverse-transcription PCR to measure mRNA levels of cytokines and other genes as shown. (n = 8 per group) (*F*) FACS analysis to quantify the ratios of CD11b^+^ DCs in hepatic CD45^+^ cells. Flow cytometric analysis to show ratios of (*G*) CD163^+^ cells, (*H*) CD206^+^ cells, (*I*) MHCII^+^ cells, (*J*) mean fluorescence intensity of CD11b, and (*K*) F4/80 in KCs or non-KC macrophages in the same mouse. Phenotypic comparison between KCs and non-KC macrophages were evaluated by a paired-samples *t* test. ∗*P* < .05, ∗∗*P* < .01, and ∗∗∗*P* < .001. ADM, adrenomedullin; Arg-1, arginase-1; bFGF, basic fibroblast growth factor; FMI, median fluorescence intensity; HGF, hepatocyte growth factor; iNOS, inducible nitric oxide synthase; Mac, macrophage; MMP, matrix metalloproteinase; PGF, placental growth factor; TGF, transforming growth factor; TNF, tumor necrosis factor; VEGF, vascular endothelial growth factor.

**Figure 6:**
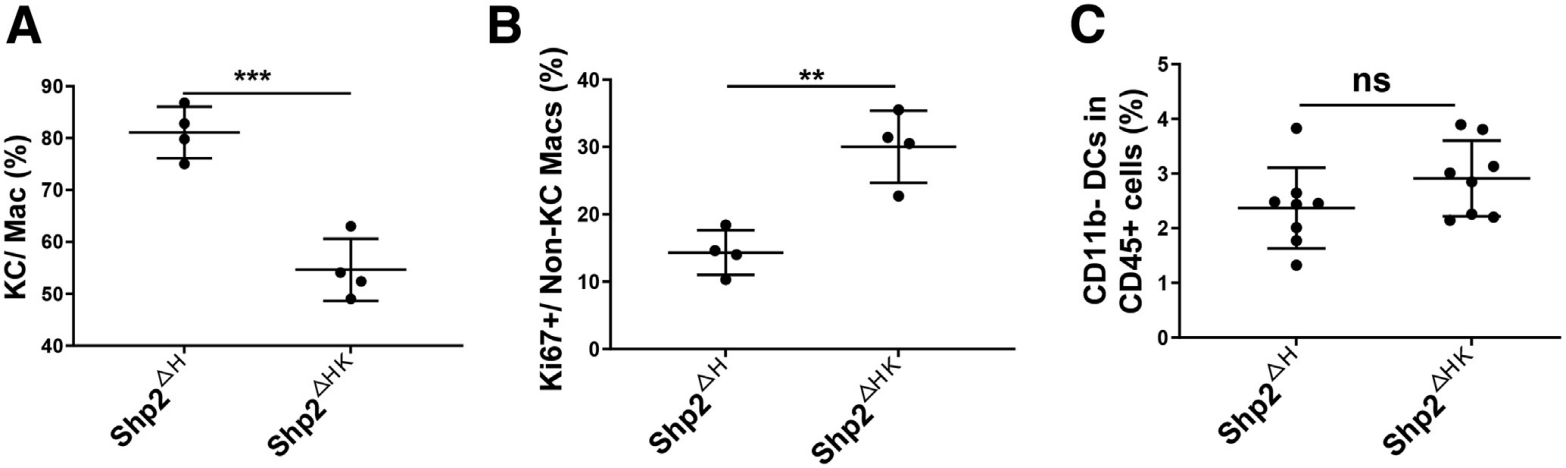
**FACS analysis to quantify ratios of related cells.** (*A*) FACS analysis to quantify ratios of KCs in total macrophages in the liver. (*B*) FACS data showing ratios of Ki67^+^ cells in non-KC macrophages in the liver. (*C*) FACS data showing ratios of CD11b^-^ DCs in CD45^+^ cells in the liver. ∗∗*P* < .01; ∗∗∗*P* < .001. Mac, macrophage.

To investigate mechanisms underlying the tumor-promoting effect in Shp2^ΔHK^ mice, we compared immune cell compositions between Shp2^ΔH^ and Shp2^ΔHK^ mice 3 weeks after transfection of Ras/Myc oncogenes. Although no significant difference was observed in total macrophages (Figure 5*A*), we detected down-regulation of the KC subpopulation with increased non-KC macrophages in Shp2^ΔHK^ mice, relative to Shp2^ΔH^ mice (Figures 5*B* and 6*A*). Immunostaining further confirmed that TAMs were Clec4f-negative, non-KC macrophages, which were expanded in Shp2^ΔHK^ liver (Figure 5*C*). TAMs are among the most abundant stromal cell types within the tumor microenvironment (TME), which are crucial drivers of tumor progression by creating an immunosuppressive microenvironment.^18^ The proliferation rate of non-KC macrophages increased significantly in Shp2^ΔHK^ liver, compared with Shp2^ΔH^ liver (Figures 5*D* and 6*B*), contributing to the expansion of TAMs. We measured mRNA levels of TAM-related cytokines, and detected significant increase of tumor necrosis factor α, MMP12, and MMP13 in Shp2^DHK^ mice (Figure 5*E*). Matrix metalloproteinases (MMPs) are zinc-dependent proteases and are involved in degradation of extracellular matrix, and represent the most prominent family of proteases associated with tumorigenesis.^19^ Although IL6 was viewed previously as a critical messenger between hepatocytes and KCs in driving liver tumorigenesis,^5^ we did not detect significant difference in IL6 expression between Shp2^ΔHK^ and Shp2^ΔH^ livers (Figure 5*E*).

**Figure 7:**
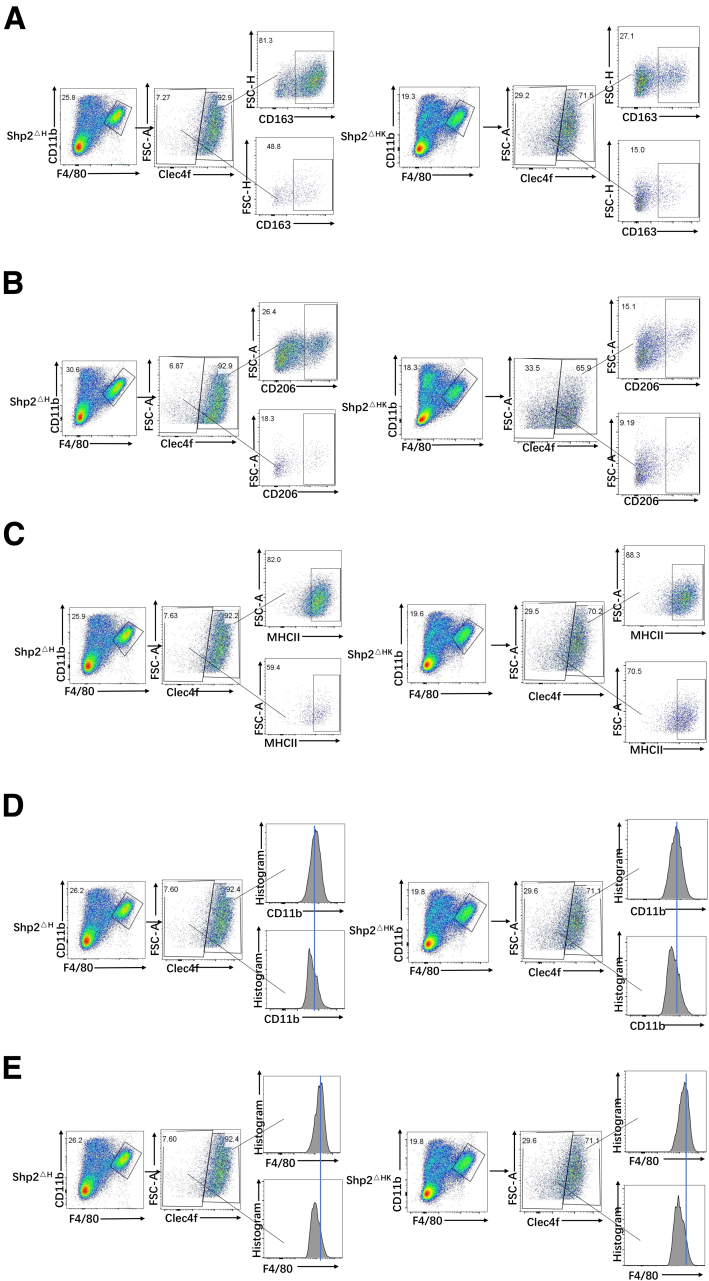
**FACS analysis to show membrane marker expression of KCs and non-KC macrophages.** Representative flow cytometric images to show expression of (*A*) CD163, (*B*) CD206, (*C*) MHCII, (*D*) CD11b, and (*E*) F4/80 in KCs and non-KC macrophages in the liver. FSC-A, forward scatter-area.

Myeloid-derived suppressor cells (MDSCs) and dendritic cells (DCs) are 2 other cell subsets differentiated from monocytes, in association with tumor progression.^20^ We observed expansion of both the MDSC and DC pools in Shp2^ΔHK^ mice (Figure 5*B*), likely contributing to the tumor-promoting effect. MDSCs are characterized by their myeloid origin, immature status, and a remarkable capacity to suppress T-cell responses.^20^ CD11b^+^ DCs were reported to suppress CD8 T cells or promote T helper 2 (Th2) cell responses to establish an immune-suppressive microenvironment.^21^^,^^22^ CD11b^+^ DCs increased in Shp2^ΔHK^ mice (Figure 5*F*), with no significant change observed in CD11b^-^ DCs (Figure 6*C*). KCs defined as Clec4f^+^CD11b^+^F4/80^high^ macrophages also are featured to highly express CD163, CD206, and major histocompatibility complex (MHC)II.^15^^,^^23^^,^^24^ Relative to KCs, the non-KC macrophages showed much lower expression for the markers CD163 (Figures 5*G* and 7*A*), CD206 (Figures 5*H* and 7*B*), MHCII (Figures 5*I* and 7*C*), CD11b (Figures 5*J* and 7*D*), and F4/80 (Figures 5*K* and 7*E*) in Shp2^ΔH^ and Shp2^ΔHK^ livers, suggesting that the non-KC macrophages were less differentiated than KCs. Meanwhile, CD163 expression in KCs was significantly higher in Shp2^ΔH^ than Shp2^ΔHK^ mice (Figures 5*G* and 7*A*), indicating that Shp2^ΔH^ mice possessed more mature KCs than Shp2^ΔHK^ mice that had more newly differentiated and less mature KCs. Thus, Shp2 deficiency in hepatocytes and KCs induced expansion of multiple myeloid cell subtypes, including TAMs, MDSCs, and CD11b^+^ DCs, collectively contributing to the HCC-promoting microenvironment in Shp2^ΔHK^ liver.

### TAM Blockade and Total Macrophage Depletion Had Opposite Effect in Tumor Progression in Shp2^ΔHK^ Mice

**Figure 8:**
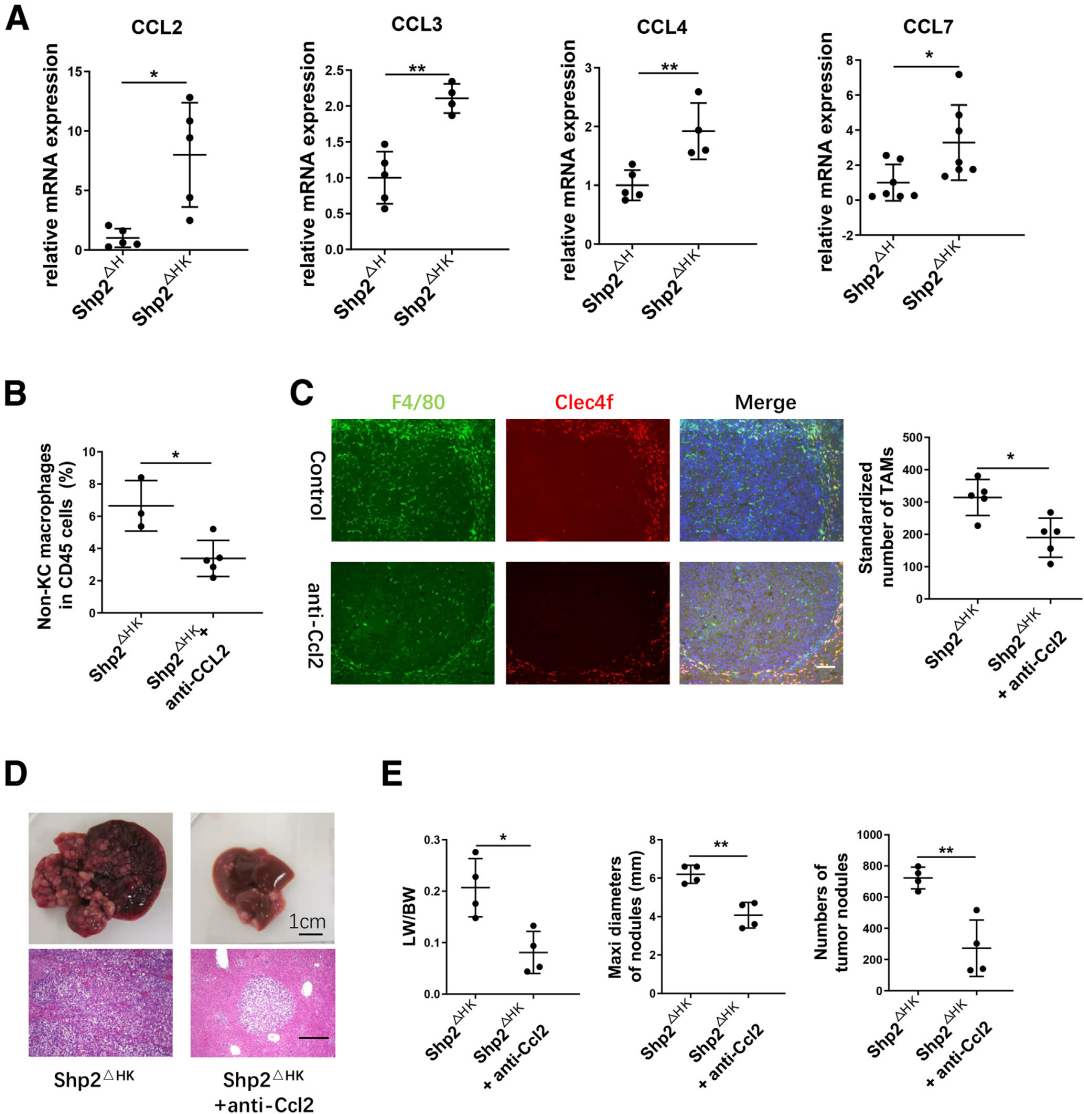
**CCL2 antibody suppresses bone marrow-derived macrophage recruitment and liver tumor progression in Shp2**^**ΔHK**^**mice.** (*A*) Quantitative reverse-transcription PCR to examine expression of chemokine genes in NPCs isolated 3 days after N-Ras/c-Myc oncogene transfection. Data are presented as means ± SD (n = 4–7). After anti-CCL2 antibody (Ab) injection as described in panel 10*A*, (*B*) a FACS analysis was performed to quantify the ratios of non-KC macrophages in hepatic CD45^+^ cells 3 weeks after oncogene transfection. (*C*) Representative immunostaining and quantification of macrophages in liver sections after anti-CCL2 antibody injection are shown. Magnification, ×100; *scale bar*: 100 μm. (*D* and *E*) Anti-CCL2 Ab or isotype IgG (200 μg) was injected intraperitoneally every 3 days starting from 1 day before oncogene transfection, all mice were killed 5 weeks after oncogene transfection. (*D*) Representative macroscopic views and H&E staining of liver sections (magnification, ×100; *scale bar*: 200 μm) are shown, and (*E*) tumor burdens were evaluated. ∗*P* < .05; ∗∗*P* < .01. LW/BW, liver weight/body weight; Maxi, maximum.

**Figure 9:**
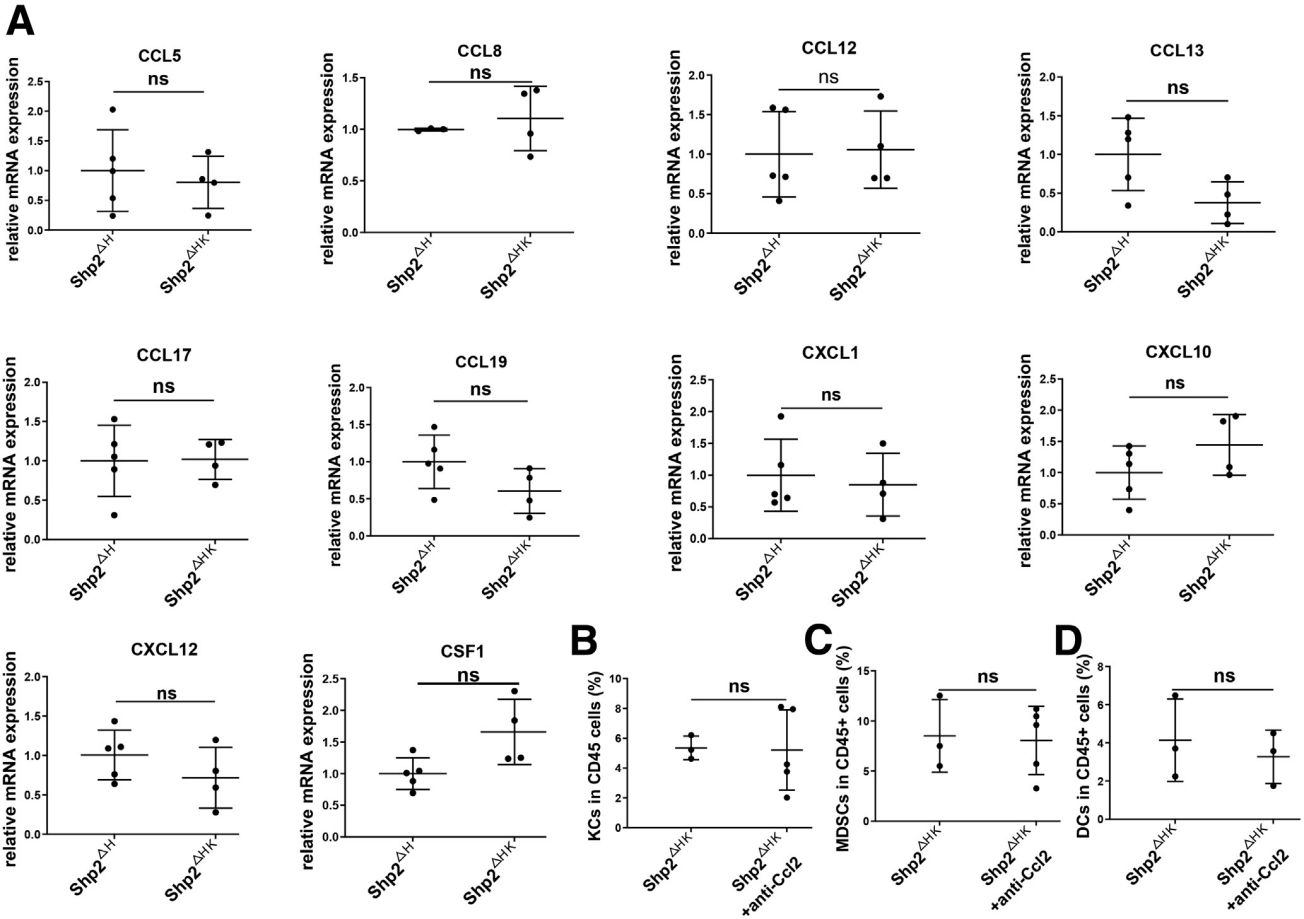
**Quantitative reverse-transcription PCR data to show mRNA levels of related chemokines.** (*A*) Relative mRNA levels of CCL5, CCL8, CXCL12, CCL13, CCL17, CCL19, CXCL1, CXCL10, CXCL12, and CSF1. (*B*) Ratios of KCs in the CD45^+^ cell pool. (*C*) Ratios of MDSCs in CD45^+^ cells. (*D*) Ratios of DCs in CD45^+^ cells. ns, no significance.

**Figure 10:**
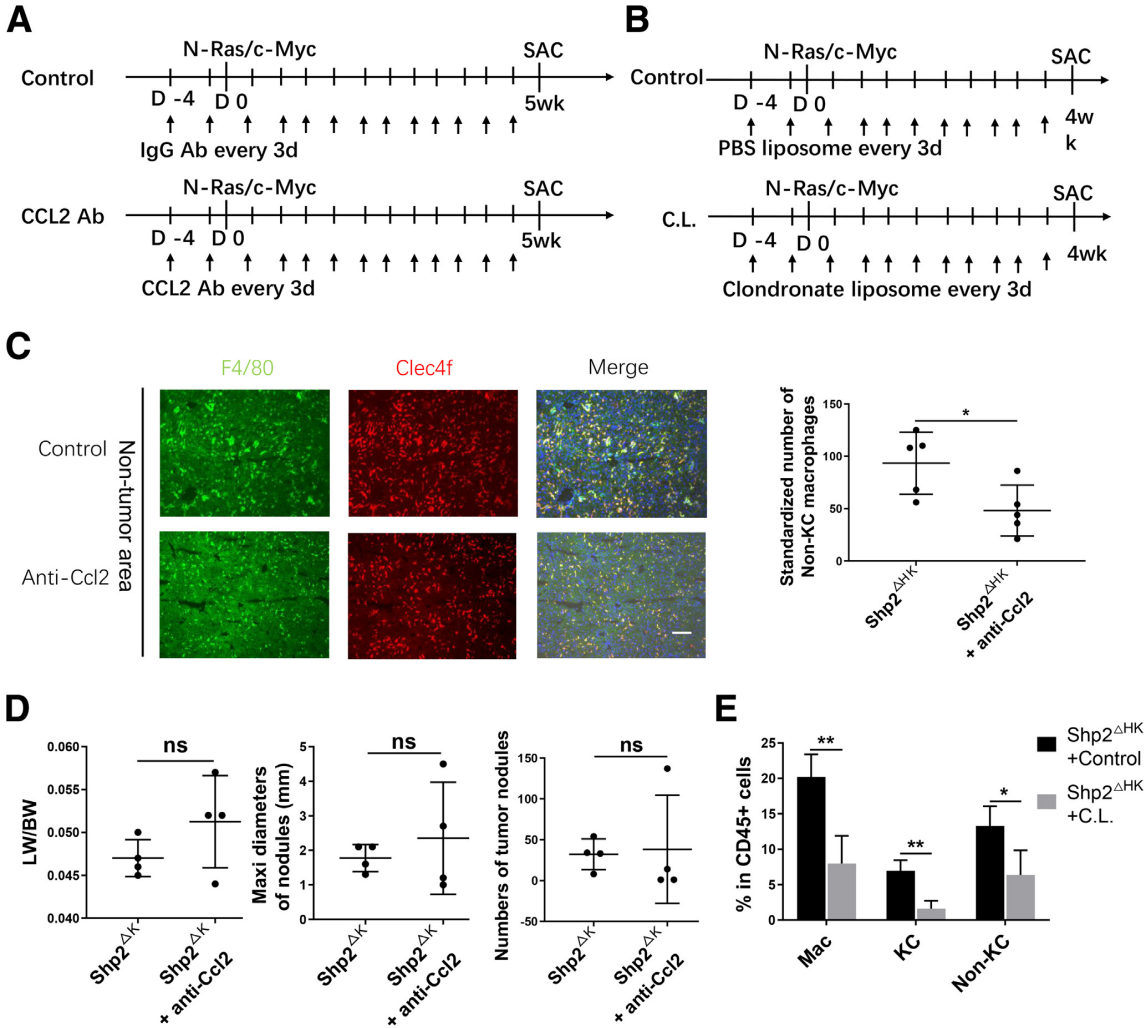
**CCL2 antibody showed no effect on liver tumor progression in Shp2**^**ΔK**^**mice.** (*A*) The experimental procedure for CCL2 antibody (Ab), or isotype IgG treatment. N-Ras/c-Myc was transfected into 2 groups of mice on day 0. CCL2 Ab or isotype IgG (200 μg/d) was injected intraperitoneally every 3 days. All mice were killed 5 weeks after oncogene transfection. (*B*) The experimental procedure for clodronate liposome, or control phosphate-buffered saline (PBS) liposome treatment. N-Ras/c-Myc was transfected into 2 groups of mice on day 0. Clodronate liposome, or control PBS liposome (200 μL/d), was injected intraperitoneally every 3 days. All mice were killed 4 weeks after oncogene transfection. (*C*) Representative immunostaining and quantification of KCs (F4/80^+^ and Clec4f^+^) and non-KC macrophages (F4/80^+^ but Clec4f^-^) in nontumor areas of the liver sections. Magnification, ×100; *scale bar*: 100 μm. (*D*) CCL2 Ab or isotype IgG (200 μg) was injected intraperitoneally every 3 days starting from 1 day before oncogene transfection, and all mice were killed 5 weeks after oncogene transfection. Tumor burdens were evaluated in Shp2^ΔK^ mice. (*E*) Flow cytometric analysis showing the ratios of the indicated cell types in CD45^+^ cells in the liver. (n = 6 per group) ∗*P* < .05; ∗∗*P* < .01. CL, clodronate liposome; LW/BW, liver weight/body weight; Mac, macrophage; Maxi, maximum; SAC, sacrifice.

In search for factors involved in recruiting bone marrow–derived monocytes/macrophages, we isolated NPCs and performed a quantitative reverse-transcription PCR analysis to examine chemokine expression. The mRNA levels of Ccl2, Ccl3, Ccl4, and Ccl7 were significantly higher in Shp2^ΔHK^ than in Shp2^ΔH^ mice (Figure 8*A*), with no significant difference observed for other chemokines between the 2 groups (Figure 9*A*). Because Ccl2 is a key chemokine known for macrophage recruitment,^25^ we tested its putative role by injecting anti-Ccl2 antibody into Shp2^ΔHK^ mice (Figure 10*A*). Indeed, administration of Ccl2 antibody suppressed recruitment of non-KC macrophages (Figure 8*B*), with no significant impact on KCs, MDSCs, and DCs (Figure 9*B–D*). Immunostaining with anti-F4/80 also showed significantly decreased TAMs after Ccl2 neutralization (Figure 8*C*), showing its efficiency for TAM blockade, and non-KC macrophages in nontumor areas also decreased after Ccl2 antibody injection (Figure 10*C*). Furthermore, Ccl2 neutralization reduced tumor burdens in Shp2^ΔHK^ mice (Figure 8*D* and *E*), with no significant effect on tumor growth in Shp2^ΔK^ mice (Figure 10*D*).

**Figure 11:**
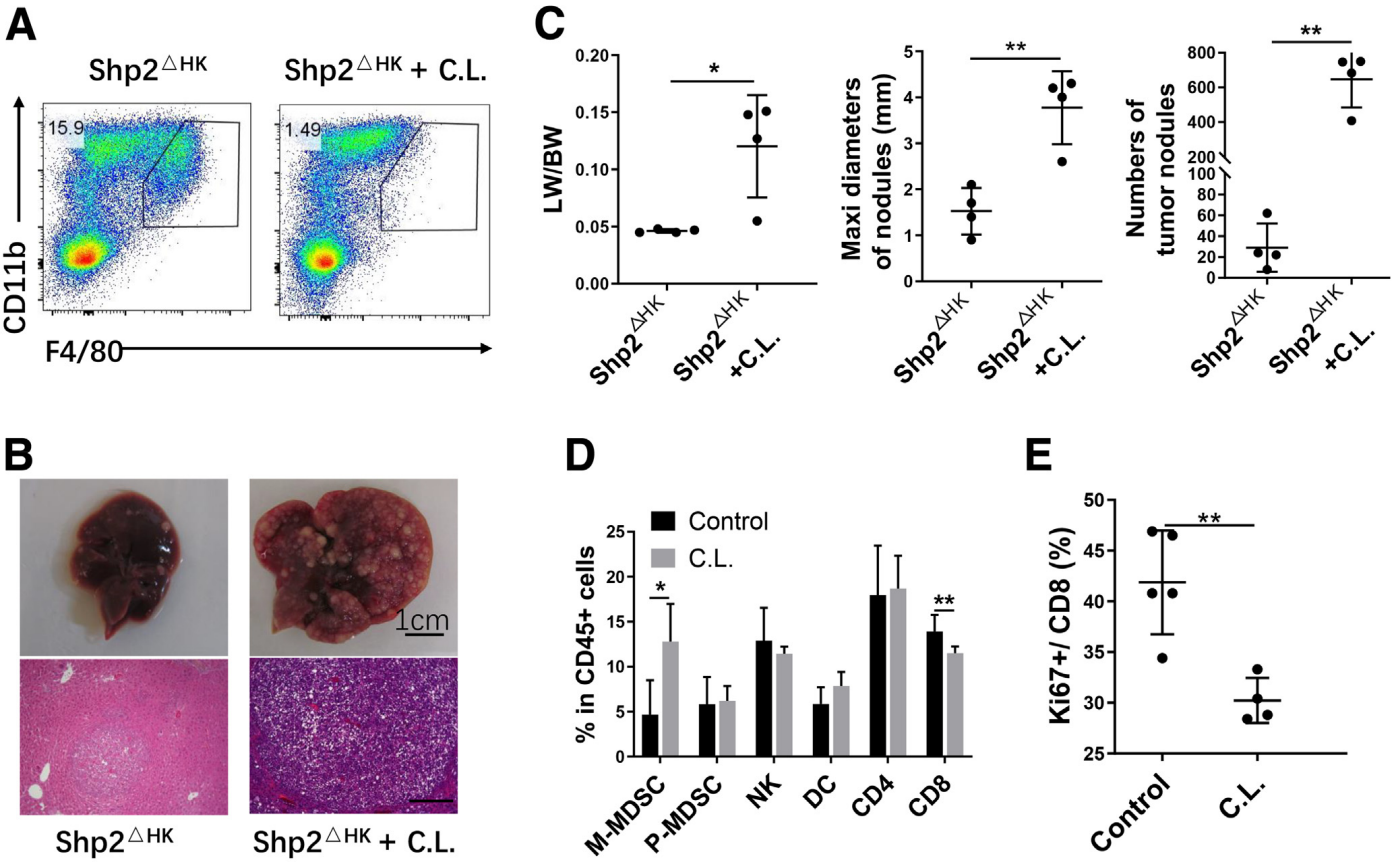
**Depleting all macrophages exacerbates tumor progression in Shp2**^**ΔHK**^**mice.** (*A*) FACS analysis of macrophages (CD11b^+^ F4/80^high^) 3 days sfter C.L. injection. (*B*) Clodronate liposome (200 μL; or phosphate-buffered saline control liposome, 200 μL) was injected intraperitoneally every 3 days starting from the day before Ras/Myc oncogene transfection, and all mice were killed 4 weeks after oncogene transfection. Representative macroscopic views and H&E staining of liver sections (magnification, ×100; *scale bar*: 200 μm) were shown. (*C*) Tumor burdens were evaluated as liver weight/body weight (LW/BW) ratios, maximum (maxi) diameters, and the number of tumor nodules. (*D*) Various immune cell ratios in hepatic CD45^+^ cells 3 days after C.L. injection. (n = 6 per group) (*E*) Ki67^+^ cell ratios in CD8 T cells. ∗*P* < .05; ∗∗*P* < .01. C.L., clodronate liposome; NK, natural killer; P-MDSC, polymorphonuclear myeloid-derived suppressor cell.

We injected clodronate liposome to deplete all macrophages (Figures 10*B* and *E* and 11*A*), and examined its impact on tumor growth. Interestingly, in contrast to the effect of Ccl2 antibody, depleting all macrophages by clodronate aggravated HCC development (Figure 11*B* and *C*). FACS analysis showed up-regulation of M-MDSC, reduced numbers of CD8 T cells (Figure 11*D*), and decreased CD8 T-cell proliferation (Figure 11*E*) after macrophage depletion. These results are consistent with a previous report that depletion of all macrophages by clodronate liposome dramatically increased liver tumorigenesis from xenografted cancer cells.^26^ Although the mechanism is not fully understood, depleting macrophages likely enhanced recruitment of monocytes into the liver, similar to DT-mediated KC depletion.^13^^,^^15^^,^^23^ In the tumor microenvironment, these newly recruited monocytes differentiated into M-MDSCs, which inhibited proliferation of CD8 T cells.^27^ Thus, selective blockade of TAMs recruitment inhibited HCC development, while depleting all macrophages induced a tumor-promoting niche in the liver.

### Depleting CD8 T Cells Accelerates and Aggravates Liver Tumorigenesis in Shp2^ΔHK^ Mice

**Figure 12:**
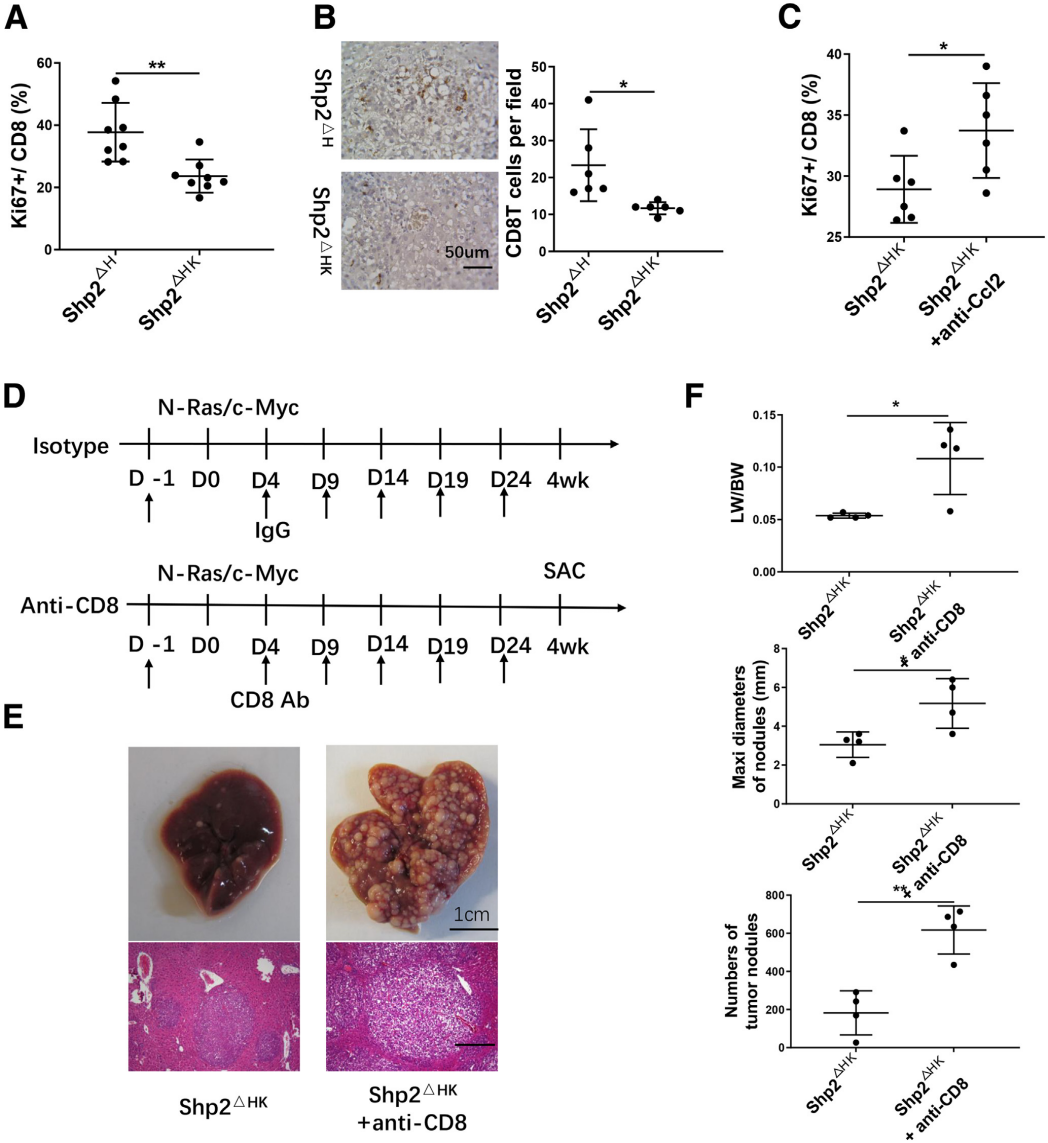
**The antitumor effect of CD8 T cells in Shp2**^**ΔHK**^**mice.** (*A*) Flow cytometric analysis to quantify Ki67^+^ cell ratios in CD8 T cells in the livers 3 weeks after Ras/Myc oncogene transfection. (*B*) Representative immunostaining and quantification of standardized CD8 T cells in tumor areas of liver sections. Magnification, ×400; *scale bar*: 50 μm. (*C*) FACS analysis to quantify Ki67^+^ cell ratios in hepatic CD8 T cells 3 weeks after anti-CCL2 antibody (Ab) injection. (*D*) The experimental scheme for injection of anti-CD8 Ab or isotype IgG. Ras/Myc transfection was performed on day 0. Antibody (200 μg/d) was injected intraperitoneally every 5 days starting from the day before oncogene transfection. All mice were killed 4 weeks after oncogene transfection. (*E*) Representative macroscopic views and H&E staining of liver sections (magnification, ×100; *scale bar*: 200 μm). (*F*) Tumor burdens were evaluated as liver weight/body weight (LW/BW), maximum (maxi) diameters, and the number of tumor nodules. ∗*P* < .05; ∗∗*P* < .01. SAC, sacrifice.

**Figure 13:**
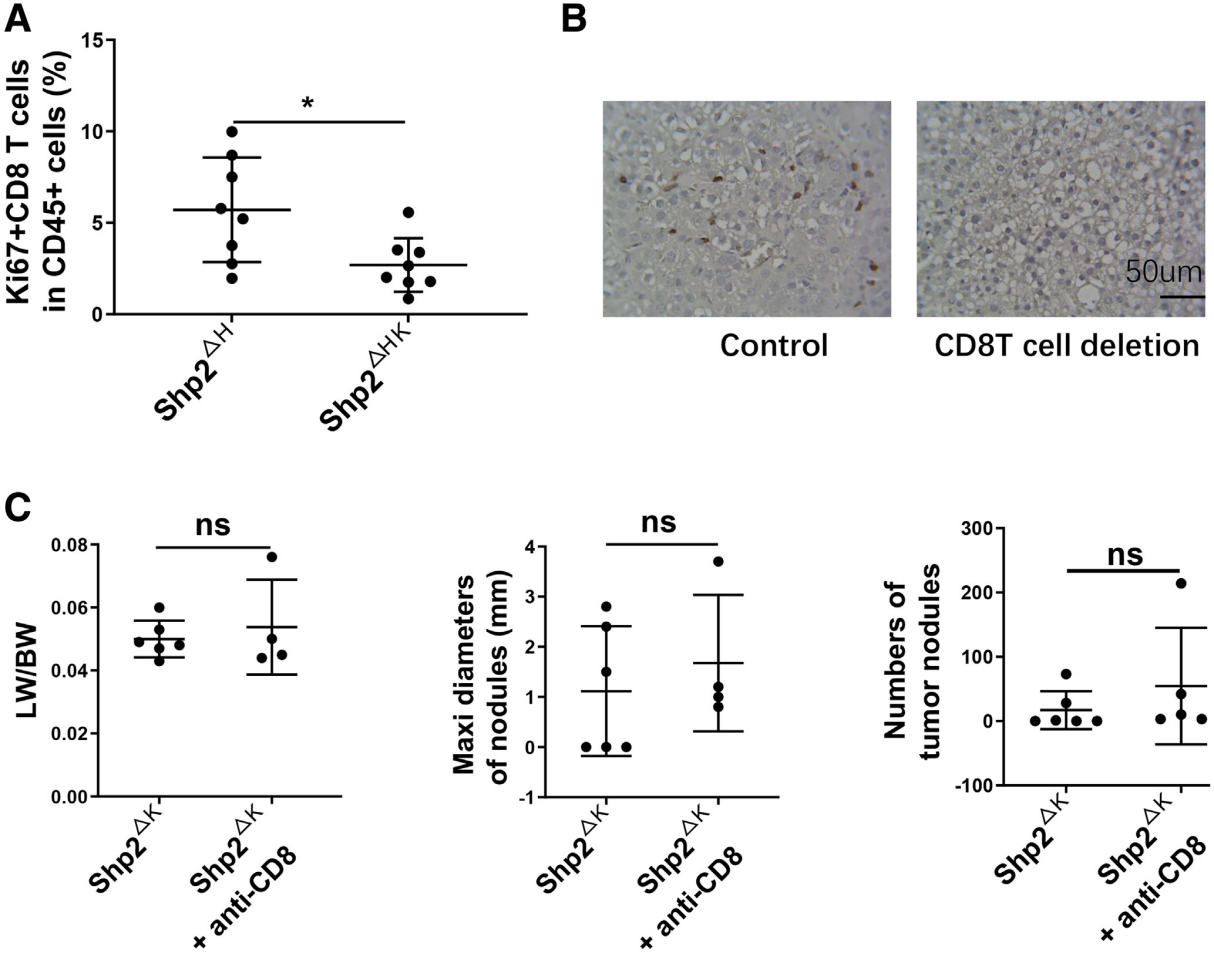
**Depletion of CD8 T cells showed no effect on liver tumor progression in Shp2**^**ΔK**^**mice.** (*A*) Flow cytometric analysis showing the ratios of proliferating (Ki67^+^) CD8 T cells in CD45^+^ cells in the liver. (*B*) Representative immunostaining to show the efficiency of CD8 T-cell deletion. (*C*) Tumor burdens were evaluated by liver weight/body weight (LW/BW), maximum (maxi) diameters, and the number of tumor nodules in Shp2^ΔK^ mice after CD8 T-cell depletion. ∗*P* < .05.

FACS analysis showed a decrease of proliferating CD8 T cells in Shp2^ΔHK^ mice 3 weeks after Ras/Myc oncogene injection, relative to Shp2^ΔH^ mice (Figures 12*A* and 13*A*), and also a significant reduction of infiltrated CD8 T-cell numbers in tumor areas in Shp2^ΔHK^ mice (Figure 12*B*). Consistent with previous reports that TAMs suppressed CD8 T-cell expansion,^18^^,^^28^ proliferating CD8 T cells indeed were up-regulated by TAM blockade with anti-CCl2 antibody injection (Figure 12*C*). These results suggest that increased TAMs inhibited CD8 T-cell proliferation and function in Shp2^ΔHK^ liver. To determine a functional role of CD8 T cells in HCC development, we injected CD8 antibody to deplete CD8 T cells (Figure 12*D*). Injecting anti-CD8 antibody at D4, 9, 14, 19, and 24 efficiently suppressed CD8 T cell numbers in the liver (Figure 13*B*). Indeed, depletion of CD8 T lymphocytes exacerbated tumorigenesis in Shp2^ΔHK^ livers when the tumor phenotype was examined 4 weeks after oncogene transfection (Figure 12*E* and *F*), with no significant effect on tumor growth in Shp2^ΔK^ mice (Figure 13*C*). Together, these results suggest that up-regulated TAMs promote HCC development in Shp2^ΔHK^ mice at least in part through down-regulation of CD8 T-cell proliferation and functions.

**Figure 14:**
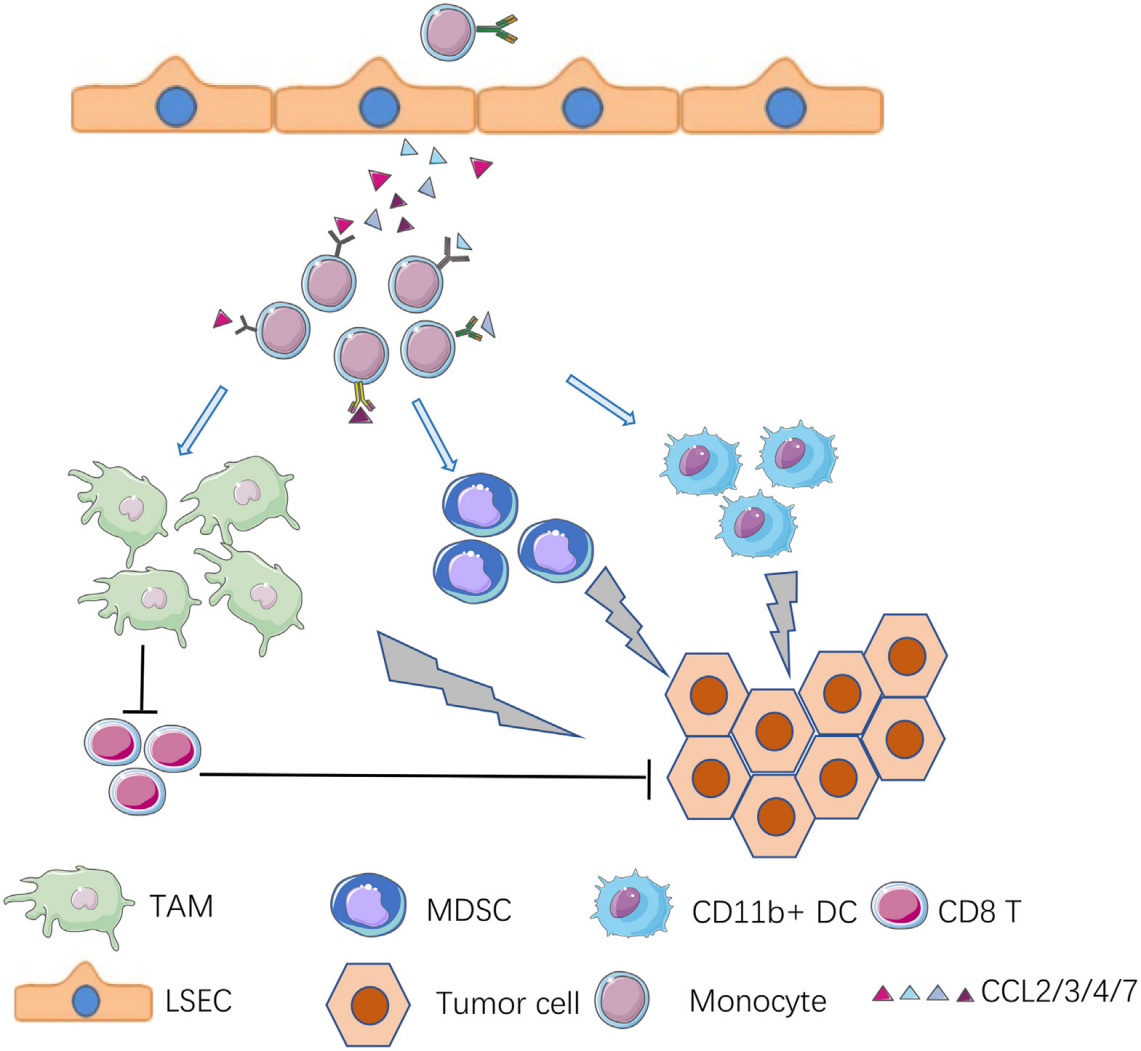
**A model for aggravated liver tumorigenesis in Shp2**^**ΔHK**^**mice.** Shp2 deficiency in KCs and hepatocytes led to significant KC down-regulation and hepatic recruitment of monocytes by chemokines CCL2/3/4/7. In tumor areas, these monocytes differentiated into TAMs, MDSCs, and CD11b^+^ DCs, thus creating a tumor-promoting microenvironment. TAMs enhanced HCC progression at least in part by inhibiting CD8 T lymphocytes. LSEC, liver sinusoidal endothelial cells.

## Discussion

Herein we present data showing that deleting Shp2, a pro-oncogenic molecule, in KCs and hepatocytes aggravated HCC development in mouse models. It has been recognized that intercellular communications between hepatocytes and NPCs, especially KCs, play complex roles in driving HCC initiation and progression, with the underlying mechanisms to be elucidated. Several groups addressed this issue by generating cell type–specific gene knockout mouse models, using Alb-cre and Mx1-cre transgenic mouse lines to delete target genes in hepatocytes and KCs.^5^^,^^6^^,^^8^^,^^9^ Their results showed opposite functions of signaling molecules in hepatocytes and KCs, with regard to HCC development. One striking phenotype is that deleting pro-oncogenic molecules in both KCs and hepatocytes suppressed HCC, using the polyIC-induced Mx1-Cre system. On the contrary, our data presented here indicate that Shp2 removal in KCs and hepatocytes, mediated by Clec4f-Cre and AAV-Cre, drastically promoted HCC development, which challenges a widely known model on hepatocyte/KC communication via a cytokine circuit.^5^

We found that deleting Shp2 in KCs generated a liver microenvironment conducive for tumor growth out of metastasized colorectal cancer cells. Of note, our previous experiments showed that Shp2 loss in hepatocytes aggravated HCC development induced by DEN or the oncogenes Ras/Myc.^10^^,^^17^ In this study, we generated a compound mutant mouse line with Shp2 selectively deleted from both KCs and hepatocytes. Strikingly, concurrent removal of Shp2 from the 2 cell types induced a tumor microenvironment that robustly promoted HCC development driven by the oncogenes Ras/Myc. Altogether, our results show that deleting Shp2 in either KCs or hepatocytes was protumorigenic, while removing Shp2 from both cell types showed an even more robust tumor-promoting effect. These observations are in sharp contrast to previous observations on the tumor-suppressing effects of ablating pro-oncogenic molecules in KCs and hepatocytes, using the Mx1-Cre mouse line.^5^ Together with our previous results showing an antitumor effect of polyIC, the inducer of Mx1-Cre expression, we believe that the reported tumor-inhibiting effect mainly was owing to the strong immunomodulatory function of polyIC, rather than deletion of these signaling molecules in KCs and hepatocytes. The experimental data clarify a confusing issue and argue against application of the Mx1-Cre system to liver cancer research. It must be indicated that polyIC-driven Mx1-Cre expression mediates a target gene deletion in almost all cell types in the liver, not restricted to hepatocytes, KCs, or stellate cells, which confounds the interpretation of experimental results obtained with this system in liver studies.

Shp2 deficiency led to decreased KC numbers in the liver and therefore triggered compensatory recruitment of bone marrow–derived monocytes into the liver, which then differentiate into non-KC macrophages with TAM function (Figure 14). Compared with KCs, these non-KC macrophages have lower expression of CD163, CD206, MHCII, CD11b, and F4/80, showing a less differentiation phenotype. When the newly generated KCs start to express Clec4f, the cre system is turned on, leading to Shp2 deletion and KC apoptosis. This dynamic loop may cause persistent recruitment of monocytes and increase non-KC macrophages. Consistently, previous studies have shown that DT-mediated depletion of liver-resident KCs generated niche availability and therefore induced engraftment of bone marrow–derived monocytes, which gave rise to self-renewing and differentiated KCs.^13^^,^^15^^,^^23^ Mechanistically, functional interactions of the notch ligand Delta like canonical notch ligand 4 (DLL4) and transforming growth factor-β secreted by liver sinusoidal endothelial cells (LSECs) with endogenous liver X receptor (LXR) ligands drive the induction and functional maintenance of KCs through up-regulation of recombination signal binding protein for immunoglobulin Kappa J Region (RBPJ) and LXRα and reprogramming of the repopulating macrophage enhancer landscape.^13^

We observed a TAM function of these non-KC macrophages by the HCC-inhibiting effect of CCL2 antibody, which effectively suppressed their recruitment into the liver. This is in agreement with previous data that TAMs were monocyte-derived macrophages. KCs are stationary cells located in the vasculature, adherent to LSECs, and exposed directly to the contents of blood.^29^ Indeed, combinatorial interactions of LSECs and KCs were required for induction and maintenance of KC identity.^13^ Thus, monocytes in tumor tissues differentiated into non-KC TAMs, instead of mature KCs. This study also showed that the expanded population of TAMs was associated with a reduction of CD8 T cells in both primary and metastatic liver tumor models (Figure 14). Functionally, TAMs in tumor-bearing livers inhibited proliferation of CD8 T cells, thus contributing to the protumorigenic effect in Shp2^DHK^ liver. It was reported previously that altered amino acid metabolism in TAMs resulted in production of arginase and immunosuppressive metabolites via the indoleamine 2,3-dioxygenase pathway responsible for metabolic starvation in T cells.^18^ Macrophages also were shown to modulate CD8 T-cell infiltration by inducing fibrosis.^30^ Targeting chemokines or its receptors to block recruitment of TAMs appears to be a promising therapeutic strategy for HCC.^25^^,^^28^^,^^31^ Interestingly, deleting Shp2 in KCs aggravated progression of liver tumors grown from metastasized colorectal cancer cells, but had no significant effect on primary tumors driven by Ras/Myc oncogenes. This could be owing to difference in antigenic properties of the tumor cells, but also may be caused by a more profound impact of the impaired innate immunity on tumor progression than tumor initiation or oncogene-induced cell transformation in Shp2^DK^ mice. Indeed, dual deletion of Shp2 in KCs and hepatocytes further down-regulated hepatic immune functions, resulting in more severe growth of tumors induced by Ras/Myc oncogenes in Shp2^DHK^ mice. This is consistent with our previous data showing that Shp2 deficiency in hepatocytes down-regulated macrophage function in clearance of liver tumor-initiating cells driven by the oncogenes in Shp2^DH^ mice.^17^

It is remarkable that depleting all macrophages by injecting clodronate liposomes promoted liver cancer development, opposite to the tumor-suppressing effect of blocking TAM recruitment by anti-CCL2 antibody. These opposing results illustrate vividly the complexity of macrophage composition with multifaceted functions in liver tumorigenesis, which raised caution on manipulating macrophages for oncological treatment. If the antitumorigenic KCs were removed, the newly recruited monocytes in the liver would differentiate into MDSCs, which can down-regulate CD8 T cells and promote liver tumorigenesis. Thus, a promising strategy in liver cancer therapy would be to eliminate TAMs specifically by targeting their unique membrane marker(s) or to block their hepatic recruitment.

Because Shp2 is currently an extremely popular drug target in the pharmaceutical industry for cancer therapy, our study on its effects in various cell types will guide the design of new therapeutic strategies based on targeting Shp2 alone or in combination. We believe that a most effective therapy must rely on cell type–specific delivery of a potent Shp2 inhibitor through nanotechnology or other means.

## Materials and Methods

### Mouse Lines and Tumor Models

The animal protocols (S09108) were approved by the Institutional Animal Care and Use Committee of the University of California San Diego, following National Institutes of Health guidelines. The Shp2^ΔK^ (Shp2^F/F^:Clec4f-Cre^+/-^) mouse line in the C57BL/6 background was generated by breeding the Shp2^F/F^ mouse with Clec4f-Cre^+/-^ transgenic mice. The SHP2^ΔH^ and Shp2^ΔHK^ mouse lines were generated by giving a single intraperitoneal injection of 2.5 × 10^11^ genome copies of AAV8-thyroxine-bindlng globulin (TBG)-Cre to WT (Shp2^F/F^) and Shp2^ΔK^ mouse lines at 4 weeks old, respectively. Mouse HCC was induced by HTVi of oncogenes together with the sleeping beauty transposase, as described. The plasmids (PT/Caggs-N-Ras-V12, PT3-EF1a-C-Myc) were gifts from Dr X. Chen at the University of California San Francisco. A total of 0.95 μg/g N-Ras and 0.05 μg/g c-Myc, together with 0.1 μg/g sleeping beauty transposase were co-transfected. The metastatic liver tumor was induced by intrasplenic injection of 4 × 10^4^ Mouse Colon Cancer Cells 38/mouse at the age of 8–10 weeks; mice were killed 16 days after transplantation.

### Isolation of NPCs and Macrophages From Liver

Liver was digested enzymatically with collagenase H (Roche) by in situ perfusion. NPCs were isolated by centrifugation at 50 × *g* for 5 minutes and were laid on top of 63% and 27% Percoll buffer. The gradients were centrifuged at 2000 relative centrifugal force (rcf) for 20 minutes at 25ºC using a SW41Ti rotor (Beckman). Macrophages were recovered from the interface between the 2 Percoll gradients, washed and cultured overnight, and then a pure population of macrophages was collected. The purity of the macrophages was determined by immunofluorescence staining and always exceeded 90%.

### Histology, Immunohistochemistry, and Immunofluorescent Assay

Liver tissue was fixed in z-Fix solution or embedded in Tissue-Tek O.C.T. Compound (Sakura Finetek) for paraffin and frozen block preparation, respectively. Paraffin sections were stained for F4/80 and CD8. Frozen tissue sections were stained for Clec4f, F4/8, CD8, and Annexin V–fluorescein isothiocyanate apoptosis assay according to the manufacturer’s procedures. The images were acquired with an Olympus IX71 microscope and CellSense software.

### Biochemical Assays and Reagents

Immunoblotting and quantitative real-time PCR were performed following standard protocols. The total RNA of NPCs was extracted with trizol reagents and reverse-transcribed using a kit. Quantitative real-time PCR was performed with master mix using the Mx3000P qPCR system (Agilent Technologies).

### Flow Cytometric Analysis

Single-cell suspensions were stained using the LIVE/DEAD Fixable Aqua Dead Cell Stain Kit first to exclude dead cells. Surface antigens then were labeled. Next, cells were permeabilized with Fix/Perm solution (Thermo Fisher Scientific) for 30 minutes at room temperature, followed by intracellular staining. Flow cytometry data were analyzed using FlowJo software (FlowJo V10). The related flow cytometry antibody sources are shown in Table 1.

**Table 1:** Key Source Table

Reagents or resources	Source	Catalog number
FACS antibodies		
Anti-mouse Granzyme B FITC	BioLegend	515403
Anti-mouse forkhead box P3 phycoerythrin	Invitrogen	12-5773-82
Anti-mouse Ki-67 pacific blue	Invitrogen	48-5698-82
Anti-mouse NK1.1 allophycocyanin	BioLegend	108710
Anti-mouse CD3e phycoerythrin-Cyanine5	eBioscience	15-0031-81
Anti-mouse CD8e phycoerythrin-Cyanine7	BioLegend	100722
Anti-mouse CD19 allophycocyanin-Cyanine7	BioLegend	115530
Anti-mouse CD4 BV605	BioLegend	100548
Anti-mouse Ly6C FITC	BioLegend	128006
Anti-mouse F4/80 pacific blue	BioLegend	123124
Anti-mouse CD11c allophycocyanin	BioLegend	117310
Anti-mouse MHCII allophycocyanin-Cyanine7	BioLegend	107628
Anti-mouse CD11b BV605	BioLegend	101257
Anti-mouse B220 BV711	BioLegend	103255
Anti-mouse CD3 FITC	BioLegend	100204
Anti-mouse CD45 peridinin chlorophyll protein complex/Cyanine5.5	BioLegend	147706
Anti-mouse F4/80 Alexa Fluor 488	BioLegend	123120
Anti-mouse Activated caspase3 phycoerythrin	BD Pharmingen	561011
Anti-mouse Clec4f- Alexa Fluor 647	BioLegend	156804
Anti-mouse CD11c allophycocyanin-Cyanine7	BioLegend	117323
Anti-mouse CD163 phycoerythrin-Cyanine7	eBioscience	25-1631-82
Antibodies for immunohistochemistry, immunofluorescence, and Western blot		
F4/80 antibody	Invitrogen	14-4801-82
CD8 antibody	Invitrogen	PA5-88265
Clec4f antibody	Thermo Fisher	PA5-47396
Shp2 antibody	Santa Cruz	sc-7384
Gapdh antibody	Cell Signaling Technologies	5172
In vivo antibodies and reagents		
Anti-mouse CD8α	Bioxcell	BE0061
Rat IgG2b	Bioxcell	BE0090
Anti-mouse CCL2	Bioxcell	BE0185
Clodronate liposome	www.liposome.com	C09T0317
PBS control liposome	www.liposome.com	P08T0317
Commercial assays		
TRIzol	Invitrogen	15596
Reverse-transcription kit	Invitrogen	4374966
Master mix	Agilent Technologies	600882
Annexin V–FITC apoptosis assay	Invitrogen	BMS500FI-100

FITC, fluorescein isothiocyanate; Gapdh, glyceraldehyde-3-phosphate dehydrogenase; PBS, phosphate-buffered saline.

### Cell Depletion and Chemokine Neutralization

For macrophage depletion, mice were injected with 200 μL clodronate liposome or 200 μL phosphate-buffered saline control liposome every 3 days. Depletion of CD8 T cells was achieved by intraperitoneal injection of 200 μg anti-mouse CD8α or 200 μg rat IgG2b as isotype control every 5 days. A total of 200 μg anti-mouse CCL2 was injected intraperitoneally every 3 days for neutralization.

### RNA-Seq and Bioinformatic Data Analysis

NPCs were isolated from liver tissues following standard protocols. Total RNAs were extracted from NPCs using the RNeasy Plus Micro Kit (#74034; Qiagen). Libraries for RNA-seq were prepared using the Illumina TruSeq v2 Kit following the manufacturer’s instructions. Sequencing was performed at the University of California San Diego Genomics Core on the NovaSeq6000 platform. Raw reads generated by RNA-seq experiments were mapped to the GRCm39 mouse reference genome using the Star program (GitHub Inc; 2.7.10a). The expression level of each gene was obtained using featureCounts v2.0.3. Differentially expressed genes were selected based on q-values (<0.1) and fold change (≥1.5). Volcano plots in figures were generated using the ggplot2 package in R.

### Statistical Analysis

Statistical analysis was performed using SPSS software version 23. Values are presented as means ± SD. Statistical significance between the means was calculated by an independent samples *t* test. Phenotypic comparison between KCs and non-KC macrophages were evaluated by a paired samples *t* test. A *P* value <.05 was considered significant.
